# Strain and Strain Recovery of Human Hair from the Nano- to the Macroscale

**DOI:** 10.3390/life13122246

**Published:** 2023-11-22

**Authors:** Brigitte Waldmann, Martin F. T. Hassler, Alexander R. M. Müllner, Stephan Puchegger, Herwig Peterlik

**Affiliations:** 1 Faculty of Physics, University of Vienna, Boltzmanngasse 5, 1090 Vienna, Austria; brigitte.waldmann@univie.ac.at (B.W.); martin.hassler@univie.ac.at (M.F.T.H.); alexander.muellner@univie.ac.at (A.R.M.M.); stephan.puchegger@univie.ac.at (S.P.); 2 Vienna Doctoral School in Physics, University of Vienna, Boltzmanngasse 5, 1090 Vienna, Austria

**Keywords:** human hair, in operandi SAXS, mechanical properties, nanostructure, hierarchical materials

## Abstract

In this study, in operandi SAXS experiments were conducted on samples of human hair with a varying degree of strain (2% within the elastic region and 10% beyond). Four different features in the SAXS patterns were evaluated: The intermediate filament distance perpendicular to and the distance from the meridional arc in the load direction, as well as the distances of the lipid bilayer peak in and perpendicular to the load direction. From the literature, one concludes that polar lipids in the cuticle are the origin of the lipid peak in the SAXS pattern, and this study shows that the observed strain in the lipids is much lower than in the intermediate filaments. We support these findings with SEM micrographs, which show that the scales in the cuticle deform much less than the cortex. The observed deformation of the intermediate filaments is very high, about 70% of the macrostrain, and the ratio of the transverse strain to the longitudinal strain at the nanoscale gives a Poisson ratio of ν_nano_ = 0.44, which is typical for soft matter. This work also finds that by varying the time period between two strain cycles, the typical strain recovery time is about 1000 min, i.e., one day. After this period, the structure is nearly identical to the initial structure, which suggests an interpretation that this is the typical time for the self-healing of hair after mechanical treatment.

## 1. Introduction

Hair is a proteinaceous fiber of mammals with several functions, including thermal insulation, mechanoreception, protection from water, and particle filtration [1,2,3,4], to point out some of many. A further property, which is particularly important for humans, is that hair has evolved from a symbol of social status to a subject of fashion, which has laid the foundation for an ever-growing cosmetics industry [5]. A strong focus of this industry is placed on coloring hair and investigating chemical aspects [6], the toxicity and health risks of these dyes [7], the use of biological dyes (e.g., from plants) in the cosmetic industry [8], and how biological hair dyes use different pore structures (in human and yak belly hair) to find their way into the material at the nanostructure level [9]. To obtain information on nanostructures, X-ray scattering has become a very important tool. The first experiments in this field are nearly 100 years old. One of the early experiments was performed by Astbury, who proposed a scheme for the assembly of the peptides in the keratin chain from X-ray scattering [10]. He also performed mechanical testing and attributed the different appearance of the X-ray patterns in the unloaded and stretched state to a transformation from α-helices to β-sheets [10,11]. Good mechanical properties are required to grow very long hairs. Stress–strain curves are the typical way to measure parameters like Young’s modulus, Poisson ratio, or failure stresses and strains. Optical methods allow for the determination of not only engineering but also true strain [12,13]. However, this method is suitable for macroscopic strain, i.e., the strain of the whole hair, but not for strain on the nanoscopic level. Moreover, the structure of hair is rather complex; it does not consist only of two structural hierarchical levels—the nano- and the macrolevel—but there are several structural features in between. A fiber being a composite with a hierarchical structure is common for many biological materials, and such phenomena are described in detail for wood, bone, and glass sponge skeletons [14]. The intriguing structure is the consequence of a long evolutionary process. All lessons learned from biological materials are not applicable immediately to the design of new technical materials [14]. However, the specific solutions nature has found have the potential for applications in finding new solutions for engineering problems [14]. The strong hierarchical organization of hair was the focus of Popescu and Höcker [5], who described hair as the most sophisticated biological composite [5] due to its hierarchical organization, its specific hydrophilic and hydrophobic interactions, and the exciting physical properties resulting from there [5].

**Figure 1:**
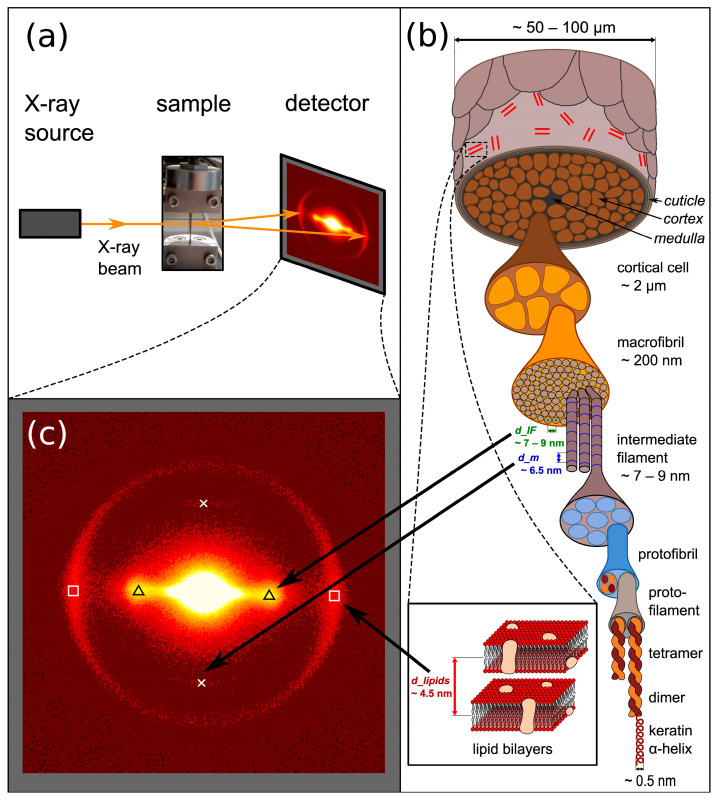
(**a**) Schematic setup of in operandi SAXS measurement, (**b**) hierarchical structure of hair from the macro- to the nanolevel, (**c**) typical SAXS pattern, enlarged from (**a**), where the important features are marked: Triangles—IF distance peak; crosses—meridional arc from molecules along the IFs; squares—ring from lipids, which was evaluated in load direction (here vertical) and perpendicular to load direction (here horizontal).

The scheme of the in operandi small-angle X-ray scattering (SAXS) measurement is shown in Figure 1a, in which X-ray patterns are recorded during the application of tension on hair samples. In Figure 1c, the SAXS pattern from Figure 1a is enlarged, and the important features are marked: triangles for the peak of the intermediate filament (IF) distance, crosses for the meridional arc of the molecules along the IFs, and squares for the ring from lipids evaluated in the direction of and perpendicular to the load. Figure 1b shows the hierarchical structure of hair, in which the structures corresponding to the SAXS features are indicated by arrows. As is visible in Figure 1b, on the basic level, hair consists of coiled-coil proteins, where four strands build up protofilaments and then protofibrils. Typically, seven to eight [5] of these protofibrils form intermediate filaments (IF), with diameters in the range of approximately 7 to 9 nm. X-ray scattering [15,16,17] and TEM [18,19] were used to describe the architecture and assembly of IFs as well as the morphological changes due to aging. As the crystallinity of the material is too low to precisely determine the arrangement of the proteins within the IFs and the packing of the IFs themselves, the sophisticated modeling of the X-ray scattering data from a synchrotron radiation source suggested initially a hexagonal 2D paracrystal IF network [20,21], and, later, a nearly uniform radial density across the IF section. An assembly of the IF into two tetrameric oligomers was proposed, in which the position of these two oligomers does not show any regularity [22]. Based on SANS data, a new model was developed with the idea of avoiding steric hindrances. Dimers assemble into a tetramer in the form of nested dumbbells (protofilaments) [23]. The protofilaments build up in the cross-section of the IFs into two main configurations: a compact ring core and a hollow ring structure [23]. On the next structural level, the IFs form macrofibrils with a size of about 200 nm, which form, on a larger scale, cortical cells in the micrometer range and, finally, the whole hair, with a typical size of 50 to 100 microns. The hair itself is not uniform in cross-section. It consists of (not always but frequently) the medulla in the center, the cortex, which has the largest proportion of the volume, and the cuticle on the outside. The latter is richer in lipids with a typical distance of 4.5 nm, which is also accessible for SAXS [9]. The lipids were described as bilayers after extraction and studying SAXS patterns as a function of water and temperature [24]. Though hair has only a diameter in the range of 50 to 100 microns, it was possible to determine the distribution of lipids across the cross-section by Fourier transform infrared synchrotron spectroscopy, from which the higher concentration of lipids inside the cuticle could be verified in different ethnic hair samples [25,26].

The mechanical properties of hair and its relationship to its structure were already investigated with X-ray scattering in the first half of the last century [10], but higher beam intensities at synchrotron radiation sources allowed in operandi investigations while continuously stretching the fibers during SAXS measurements to avoid a structural relaxation process [21]. In two different humidity conditions (45% RH and in water), the authors measured the shift of the 6.7 nm meridional arc and related this strain at the nanolevel to the macrostrain of the whole keratin sample [21]. Additionally, the center-to-center distance and radius of IFs in the paracrystal model depend on the applied macroscopic strain [21]. This pioneering paper [21] was the starting point for our investigations: our first intention was to determine all features in the X-ray pattern, which could be measured with sufficient resolution within a reasonable time, and to compare the strain at the nanoscale of these different structural elements with the macroscopic strain of human hair. From there, one can identify the amount of strain placed upon each structural element indicated in Figure 1b. This work is motivated by similar research on tendons and bones, where the nanostrain of collagen fibrils in tendons is about 40% of the macrostrain [27]. In bone, during initial elastic deformation, the ratio of fibril- to macrostrain is also about 0.4, whereas the ratio of mineral- to macrostrain is smaller, only about 0.16 in wet and 0.24 in dry samples [28]. Both strain ratios, however, start to differ, or the strain levels saturate after crossing the elastic/inelastic transition [28,29]. This indicates a redistribution of strain energy within bone tissue [28] and is a striking example of the complex deformation behavior of biological composites. In human hair, macroscopic and microscopic deformation have a common tendency, but they are also different at the macro- and nanoscale [21]. It was stated by the authors that macrofibril slippage cannot fully explain these differences in deformation. A focus was placed on the meridional arc, which arises from staggered molecules along the IFs [21].

After relating the nanoscopic to the macroscopic strain, our second goal was to look at strain relaxation and strain recovery and to find out what happens at the nanoscale. Again, this was motivated by earlier results on the self-healing of bone [30,31,32] but also on the self-healing of polymers [33,34]. In contrast to self-healing on the macroscale, which is a process on the cell level, the self-healing of bone on the nanoscale arises from sacrificial bonds [30], which break and recombine. The process involved in the plastic deformation is localized to 1 nm^3^ and has an energy in the order of 1 eV [31]. To determine the timescale of this process, in operandi SAXS was used to determine the strain relaxation in collagen and mineral fibrils. However, after fracture, the strain in the minerals is immediately released, and stress relaxation in collagen has a relaxation time of 75 s [32]. This is seen as the stress transfer time from mineral to collagen and, thus, as the characteristic time for self-healing in bone. Self-healing processes are not restricted to biomaterials such as bone. They are also a research field in technical polymers [33,34]. It should also be noted that these self-healing processes are just physical, i.e., they do not require a living tissue with cell remodeling. This is fundamental, as hair is also not a living tissue, being produced by hair follicles but composed of dead keratinocytes that are compacted into a fiber [35]. Therefore, to investigate self-healing in hair, we used in operandi SAXS and followed the strain recovery at the nanoscopic level. Though strain recovery is not identical to self-healing, the determination of the strain at the nanoscale by performing a load program of repeated load/macrostrain cycles with different waiting times can underpin the view that there are physical reconstruction processes at least similar to self-healing processes. The particular advantage of in operandi SAXS is that, on the one hand, one is able to follow structural changes at the nanoscale in operandi, and on the other hand, one can distinguish the different structural elements and their specific deformation behavior.

## 2. Materials and Methods

In operandi SAXS experiments were performed using a Nanostar (Bruker AXS, Karlsruhe, Germany), equipped with a microfocus tube (IμS high brilliance, Incoatec, Geesthacht, Germany, CuK_α_ radiation with a wavelength of λ = 0.1542 nm), a pinhole camera delivering a beam of approximately 0.5 mm diameter, and a 2D position-sensitive detector (VÅNTEC 2000 from Bruker AXS, Karlsruhe, Germany). With the sample-to-detector distance of 1.08 m, a q-range from 0.085 nm^−1^ to 2.8 nm^−1^ was accessible. All tensile test experiments were performed in a vacuum to ensure the same conditions while straining. A more precise description of the equipment and data evaluation is shown in Figures S1–S5 in the Supporting Information.

Six samples of Caucasian hair from a healthy donor without any pre-treatment such as coloring or bleaching were taken from the medium section of the hair shaft, glued on a holder made of paper with a gauge length of 15 mm, and the position of the crosshead was set to specific points corresponding to either 2% or 10% strain. Each sample used for the test consisted of 30 strands of hair, as this was sufficient for a short SAXS measurement time of 600 s for each image. This keeps relaxation effects low during the time required to take a SAXS pattern. In operandi single-hair experiments would require the intensity of an X-ray beam of a synchrotron radiation source. These experiments would have several advantages, such as a better-characterized stress distribution in the single hair, no alignment problems, and excellent precision of SAXS data, but laboratory experiments such as ours do have their merits, e.g., fewer problems with radiation damage, short measurement time, and a statistical average of different hair in one experiment.

**Figure 2:**
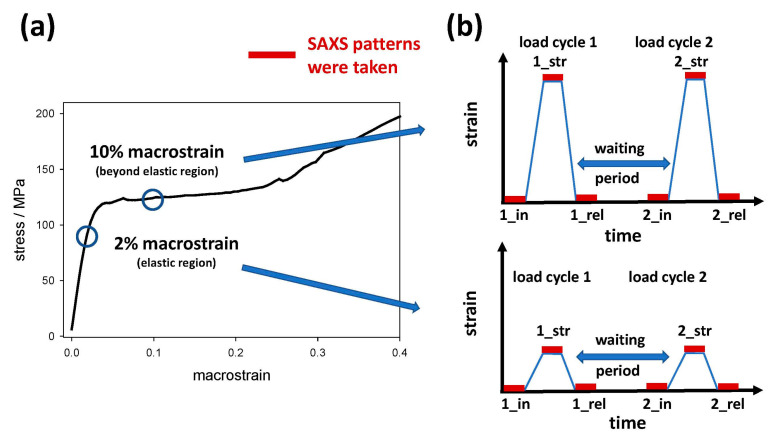
(**a**) Scheme of the measurement program: SAXS patterns were taken at fixed macroscopic strains of either 2% within the elastic region or 10% beyond, drawn as circles into a typical stress–strain curve from a hair sample. (**b**) For strain recovery, a load/strain program was chosen with a first strain cycle denoted by number 1 and the indices _in for the initial part of the experiment (zero load and strain), _str for maximum strain (either 2% or 10% macrostrain) and _rel for returning to zero strain, i.e., relief of the strain, which is followed after a certain waiting period by a second load/strain cycle with number 2 and the same indices as above. The waiting periods were chosen in logarithmic steps of 10^1^, 10^2^, 10^3^, and 10^4^ min. At each step of the strain cycle, a SAXS pattern was taken, visualized by red bars.

A typical strain cycle is shown in Figure 2b and consists of three points, where a SAXS pattern was taken, described by the index _in for the initial strain (zero strain at the beginning of the experiment), the index _str for the maximum strain and index _rel for the last SAXS pattern after returning to zero load, i.e., relief of the load and strain. After a specific waiting period at zero strain, which was chosen in decadic logarithmic steps of 10^1^, 10^2^, 10^3^, and 10^4^ min, a second cycle followed with index 2 and the same naming scheme as above; see Figure 2b. For the maximum strain, we decided to use two different values, a strain of 2% (in the elastic region) and 10% (beyond the elastic region), as is visible from the typical stress–strain curve depicted in Figure 2a. At each of these strain steps, a SAXS pattern was recorded, visible as red bars in Figure 2b. SAXS data were background corrected and radially averaged to obtain scattering intensities as a function of the scattering vector *q =* 4 *π* (sin*θ*)/*λ*, where *λ* is the X-ray wavelength and 2*θ* is the scattering angle. Particular attention was paid to precisely determining the peak maxima and peak shape, as this is important to follow even small changes in distances after the strain increased. For the lateral IF peak, a skewed normal distribution was used, the lipid ring was fitted with a Lorentzian function, and for the meridional arc from the axial staggering of molecules, the center of gravity in an interval symmetric around the intensity maximum was appropriate. The distances in real space were calculated from the peak maxima *q*_max_ in reciprocal space, *d =* 2*π/q*_max_. A distance distribution influences this relationship, but as we were interested in relative changes in each peak, we kept this procedure throughout the paper.

Scanning electron microscopy (SEM) micrographs were made with a Zeiss Supra 55 VP (Zeiss, Oberkochen, Germany). To avoid charging, hair samples were coated with 10 nm Au. Different samples were prepared to take pictures of hair subjected to the same strain conditions as the samples in the SAXS measurements, i.e., images were taken of unstrained natural hair, of hair strained by 10%, as well as of hair released for a certain waiting period and of hair strained a second time after the waiting period. For achieving and preserving strain conditions in the SEM, special sample holders were constructed, in which the hair can be fixed in its strain state.

## 3. Results

**Figure 3:**
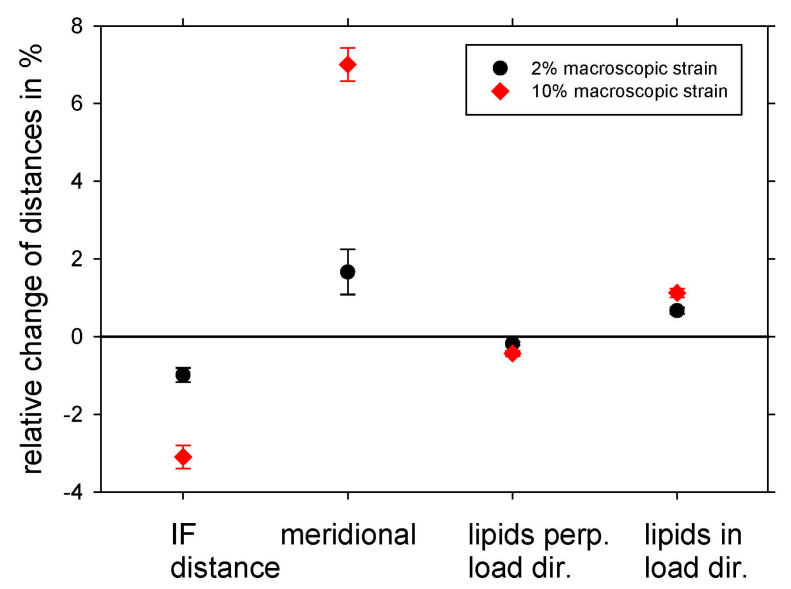
Mean distance change $\bar{{∆d/d}_{0}}$ of different structural elements evaluated from SAXS, divided by its initial value. This corresponds to the strain at the nanoscale and is shown as black circles for 2% macroscopic strain in the elastic range and as red diamonds for 10% macroscopic strain beyond the elastic range. The distance change evaluated from the meridional arc in load direction (length axis of the IFs) for 10% macroscopic strain shows the highest normalized distance change, i.e., a nanostrain of about 70% of the macroscopic strain, whereas the IF distance perpendicular to the load direction is smaller and shrinks with increasing strain. The distances from the lipid bilayers increase in load direction and decrease perpendicular to the load direction and show much lower strain values, which indicates that lipids deform to a much smaller amount than IFs.

As mentioned in the introduction, typical values for the ratio of nano- to macrostrain are about 0.4 for bone and collagen [27,28], whereas, using the meridional arc by in situ X-ray scattering in a synchrotron radiation source, a much higher value was obtained for keratin [21]. Therefore, we evaluated four different reflections: the IF distance peak, the meridional arc, and the lipid ring in and perpendicular to the load direction. Figure 3 shows the relation of nano- to macrostrain for these four reflections at 2% and 10% macroscopic strain, i.e., in the elastic region and beyond, as black circles and red diamonds, respectively. The precise numerical values for Figure 3 are found in Table 1. The standard error of the mean distances shown is only the statistical error from the initial unstrained hair bundles and not the absolute error, which is considerably larger. However, as we follow the identical hair bundle in dependence on strain and time, this relative error is crucial for the precision of the measurement. The highest value is obtained for the nanostrain from the meridional arc, i.e., the strain along the IF filaments in load direction. It is nearly 70% of the macrostrain, which means that more than two thirds of the macrostrain is also present at the nano level. Though hair has several hierarchical levels in its structure, as visualized in Figure 1b, macro- and microstrain differ much less as in collagen and bone. From this, one can conclude that the IFs are a much more dominant structural element than the collagen molecules. If IFs are strained, their length increases, but their distance decreases, visible by the opposite sign of the relative change in the IF distance peak in comparison to the one of the meridional arcs. The ratio of the nanostrain perpendicular to the load direction (obtained from the IF distance peak) to the nanostrain in load direction (obtained from the meridional arc) can be seen as a “nanoscopic Poisson ratio ν_nano_”, with a value of ν_nano_ = 3.1/7.0 = 0.44 calculated from Table 1. This high Poisson ratio, close to 0.5, is typical for materials such as soft matter and elastomers, which mostly maintain their volume under load [36], as well as for a variety of fiber networks [37]. For the calculation, the 10% macroscopic strain values are used, as the difference is larger at higher strains, and the precision, therefore, is higher. In general, the strain in the IFs scales with the macroscopic strain both in and perpendicular to the load direction in the elastic region and beyond. A significant difference is observed for the lipids, where the deformation is considerably smaller. The relative change in lipid bilayer distances is only about 1% in the load direction, even for a macroscopic strain of 10%. As lipids are mainly located in the outer parts of the hair, the cuticle, and the scales, this indicates that these parts of the hair do not take part in straining to the same amount as the inner part, the cortex, consisting mainly of IFs and matrix.

**Figure 4:**
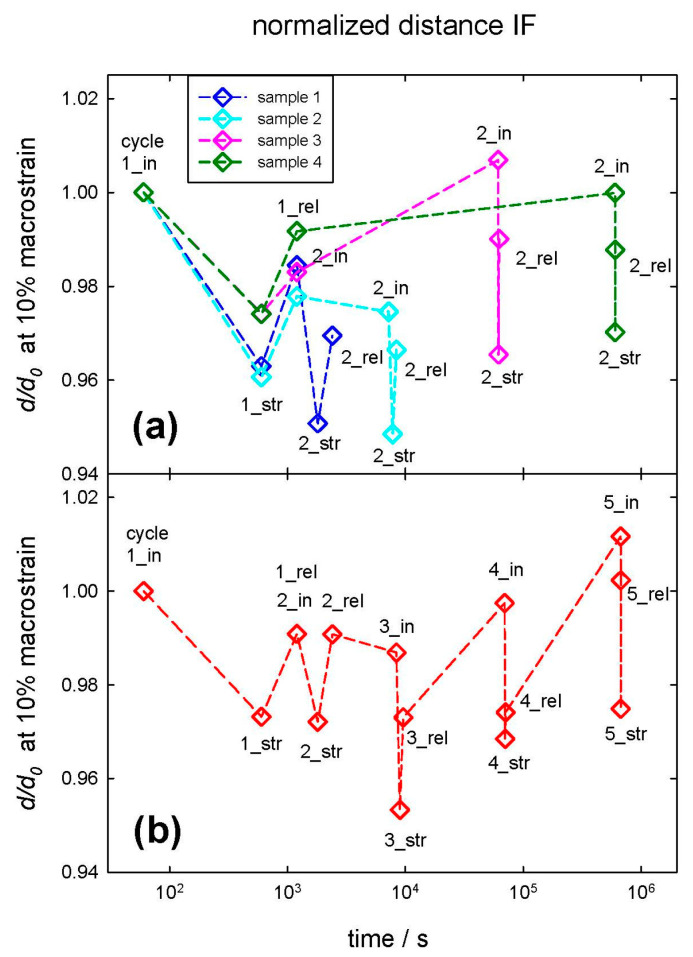
Change in distances between IFs, normalized to an initial value. Upper figure (**a**) multiple samples, a first strain cycle (with 1_in, initial distance, 1_str, distance at 10% macrostrain, 1_rel, distance after strain relief to zero) was followed by a second one (denoted by 2_in, initial, 2_str, at maximum strain, 2_rel, after strain relief to zero) after a specific waiting time (10 min, 10^2^ min, 10^3^ min, 10^4^ min). Lower figure (**b**) one sample with multiple strain cycles. The number indicates the strain cycle (2 after 10 min, 3 after 10^2^ min, 4 after 10^3^ min, and 5 after 10^4^ min). The subscripts _in, _str, and _rel are identical to (**a**). The dashed lines are for better visualization of the samples and strain cycles.

For the second question, whether strain recovery is observable in hair, we investigated the structural development cycles at the nanoscale before, between, and after successive load/strain cycles while additionally varying the waiting period between these cycles. As depicted in Figure 4a, samples were subjected to an initial strain cycle with a macrostrain of 10%, and the distances of the IFs (normalized to the initial value) were determined, denoted by 1_in for the initial distance, 1_str for the one strained to 10% macrostrain, and 1_rel for the one after strain relief to zero. The maximum strain is applied for 600 s, the time to take a SAXS pattern. Then, after a specific waiting period, a second strain cycle was performed with the same notation as described above (2_in, 2_str, and 2_rel for normalized distances initially, at 10% macrostrain and after strain relief, respectively). The time between these two strain cycles was—chosen in logarithmic steps—10^1^ min for Sample 1 (blue symbols), 10^2^ min for Sample 2 (cyan symbols), 10^3^ min for Sample 3 (pink symbols), and 10^4^ min for Sample 4 (green symbols), i.e., some minutes, an hour, a day, and a week. The decadic steps were chosen in minutes for convenience (as the measurement time of one SAXS pattern is 10 min), but in the figures, data are presented in SI-unit seconds. The dashed lines serve for better visualization of the respective set of data points for each strain cycle. The tensile load is acting along the hair axis and thus along the IFs, whereas the distance between IFs is oriented perpendicular to the load. Thus, the IF length increases whereas the IF distance decreases, i.e., the IF distance shrinks with increasing load and macrostrain. There is a considerable plasticity effect immediately after unloading, i.e., the distances after returning to zero strain (with indices 1_rel and 2_rel) differ from the initial ones (with indices 1_in and 2_in). The distance between two strain cycles (in Figure 4a between 1_rel and 2_in) remains constant for Samples 1 and 2 with a waiting time period of 10 and 100 min. For Samples 3 and 4, with a waiting time period of 1000 and 10,000 min, the distance relaxes to its original value—for these samples, the IF distances 1_in and 2_in are constant. After strain relief of the first strain cycle, the structural feature (the IF distance) is completely unaffected for the samples with a long waiting period, and complete strain recovery is observed. Thus, as strain recovery occurs between Sample 2 (with 100 min waiting period) and Sample 3 (with 1000 min waiting period), one concludes that self-healing occurs with a timescale between 100 and 1000 min, i.e., within approximately a day.

To clarify the second question, if more than one strain cycle changes the sample properties, a second experiment was performed. One sample was subjected to multiple strain cycles with identical waiting periods, as described above in Figure 4a. After an initial strain cycle, others followed after 10^1^, 10^2^, 10^3^, and 10^4^ min, respectively. This is shown in Figure 4b, in which the numbers one to five indicate the successive strain cycles, and the indices _in, _str, and _rel indicate, respectively, the initial normalized distance, the distance at maximum strain, and the distance after strain relief for each strain cycle. The data are very similar to Figure 4a, and again, one can conclude that even for more strain cycles, structural recovery takes place and requires a time between 100 and 1000 min, i.e., approximately between one hour and a day.

**Figure 5:**
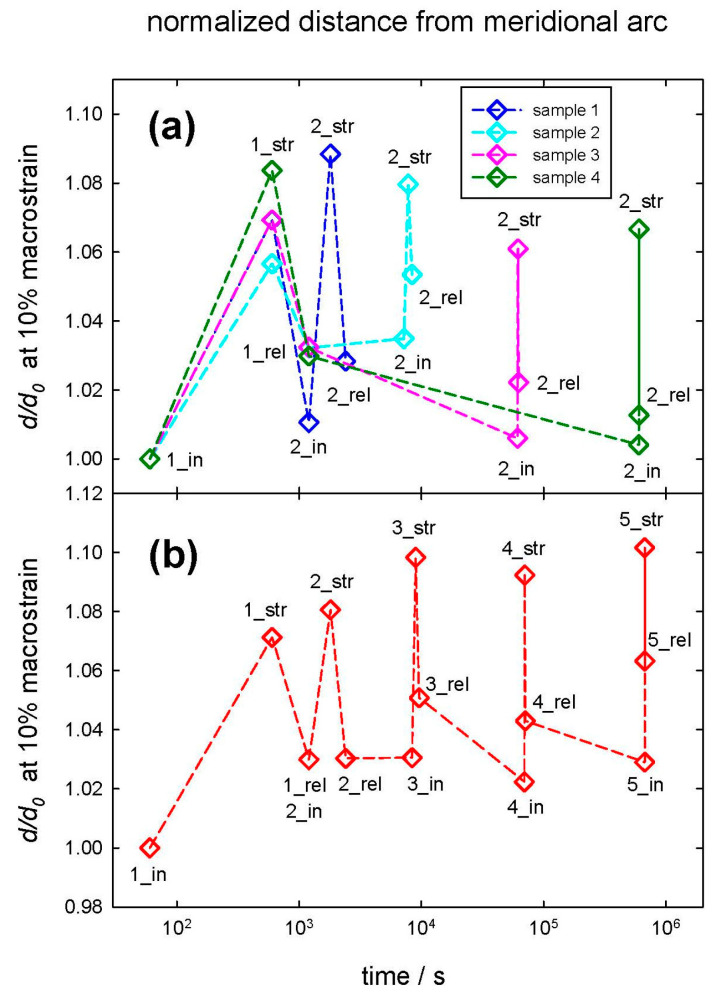
Change in IF length (evaluated from meridional arc), normalized to initial value. Upper figure (**a**) multiple samples, a first strain cycle (with 1_in, initial distance, 1_str, distance at 10% macrostrain, 1_rel, distance after strain relief to zero) was followed by a second one (denoted by 2_in, initial, 2_str, at maximum strain, 2_rel, after strain relief to zero) after a specific waiting time (10 min, 10^2^ min, 10^3^ min, 10^4^ min). Lower figure (**b**) one sample with multiple strain cycles. The number indicates the strain cycle (2 after 10 min, 3 after 10^2^ min, 4 after 10^3^ min, and 5 after 10^4^ min); the subscripts _in, _str, and _rel are identical to (**a**). The dashed lines are for better visualization of the samples and strain cycles.

Although the typical distance between IFs is recovered after this typical waiting period, a strain recovery is visible but less pronounced in the length direction of the IFs. This is also the case for the 2% macrostrain data; see Supporting Information Figures S6 and S7. Figure 5 shows the data for the normalized distance change (the strain from the meridional arc for 10% macrostrain) along the IFs in the load direction, corresponding to Figure 4, or the normalized distance change in the IFs perpendicular to the load direction. There is certainly a plasticity effect that the normalized IF length (distance from the meridional arc) differs before (index _in) and after (index _rel) loading, i.e., a part of the strain is not recovered immediately after relief of the load/strain. However, from Figure 5, one can conclude that the length of the IFs, determined from the distance of the staggered molecules, also returns to its initial unstrained value within a typical time between 100 and 1000 min, i.e., approximately between one hour and a day. This effect is smaller in load direction (from the meridional arc along the IF length) as perpendicular to the load (from the IF distance), but still strain recovery is present.

**Figure 6:**
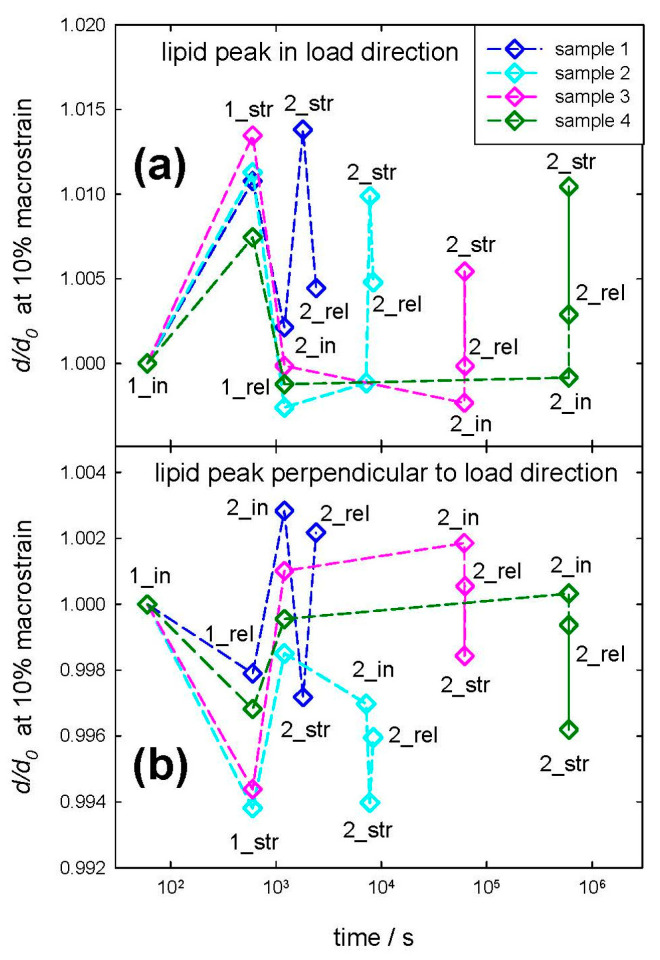
Change in lipid bilayer distance (**a**) in load direction (upper figure) and (**b**) perpendicular to load direction (lower figure). Both distances are normalized to initial value. A first strain cycle (with 1_in, initial distance, 1_str, distance at 10% macrostrain, 1_rel, distance after strain relief to zero) was followed by a second one (denoted by 2_in, initial, 2_str, at maximum strain, 2_rel, after strain relief to zero) after a specific waiting time (10 min, 10^2^ min, 10^3^ min, 10^4^ min). The dashed lines are for better visualization of the samples and strain cycles.

In Figure 6, the distance changes of the lipid bilayers are shown in the upper figure in the load direction and, in the lower figure, perpendicular to the load direction. It is natural that in the load direction, there is an extension at maximum strain for each sample or load/strain cycle, where a shrinkage is observed perpendicular to the load direction. Unusually, the value of the deformation is very low, with less than 2% in load direction and considerably less than 1% perpendicular to it, even though a macroscopic strain of 10% was applied. This means that the lipids do not take part in the strain to the same amount as the IFs. However, when calculating a Poisson ratio from the transverse to the longitudinal strain, the value is ν_lipids_ = 0.40 ± 0.03 (standard error from 13 single evaluated strain cycles), which is again typical for soft matter or elastomers. A similar result was found for straining one sample multiple times, which is found in the Supporting Information for 2% and 10% macroscopic strain; see Figures S8 and S9.

**Figure 7:**
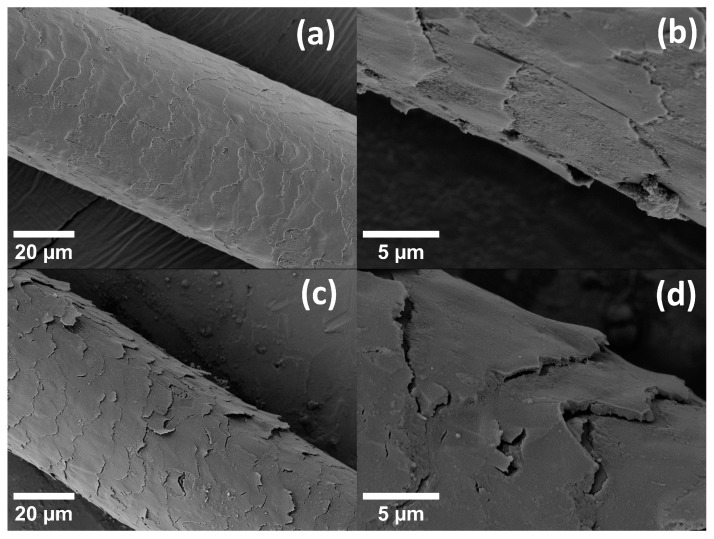
SEM micrographs showing hair initially before loading, (**a**) an overview and (**b**) in detail, and, in the lower figures, hair being subjected to a macroscopic strain of 10%, (**c**) an overview and (**d**) in detail. Scales are clearly aligned with the hair in the unstrained case and deformed and protruding from the hair shaft in the strained one.

**Figure 8:**
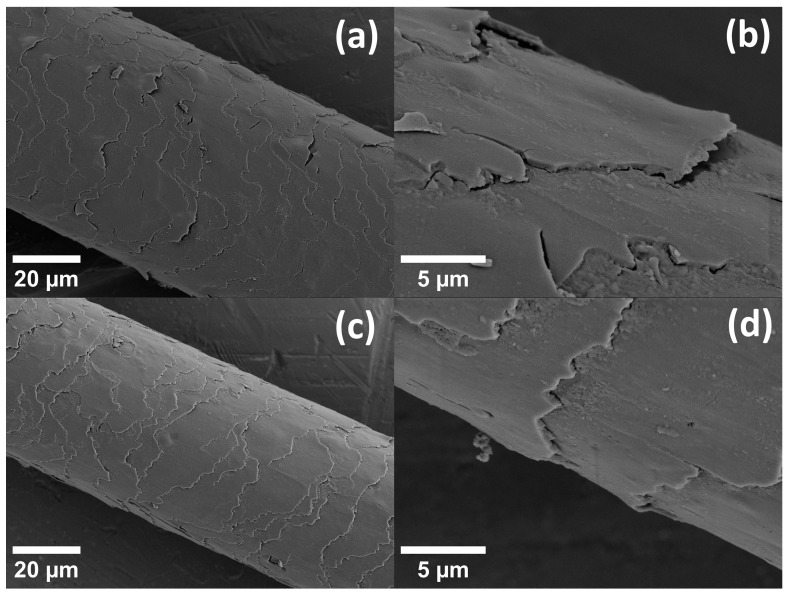
SEM micrographs showing hair after being subjected to a macroscopic strain of 10% in the upper figures after a waiting period of 100 min, (**a**) an overview and (**b**) in detail, and, in the lower figures, after a waiting period of 1000 min, (**c**) an overview and (**d**) in detail. Scales are still deformed and protruding from the hair shaft after the shorter waiting period of 100 min and nearly completely aligned again with the hair shaft after the longer waiting period of 1000 min.

In Figure 3, Figure 4, Figure 5 and Figure 6, results for the nanoscale deformation were shown. SEM micrographs show—as hair is a hierarchically structured material [5]—the deformation on the micrometer scale. Therefore, SEM micrographs of additional samples, which were subjected to the same tensile strain as in the procedure for the in operandi SAXS experiments, are presented in Figure 7 and Figure 8. Samples of hair with a gauge length of 15 mm were subjected to a strain of 10%. These samples were then fixed in specially designed sample holders that kept the hair in its current straining condition (hair was secured with glue and an annular clamp). Different samples were prepared to evaluate both natural hair (unstrained), strained hair (fixed after straining and maintaining this strained position for 10 min in a vacuum—in accordance with the time each SAXS measurement lasted), the relaxation after different waiting periods as well as the effect of repeated straining after certain waiting periods. Since SAXS measurements had shown that relaxation seems to occur between a period of 100 min and 1000 min, these two waiting periods were chosen for SEM samples between the two straining cycles. For each of these waiting periods, two samples were prepared: One was fixed in a relieved position after the waiting period had passed, and one was strained a second time and kept in a strained position for 10 min (simulating the time necessary for the SAXS measurement) and then fixed in a strained position.

**Figure 9:**
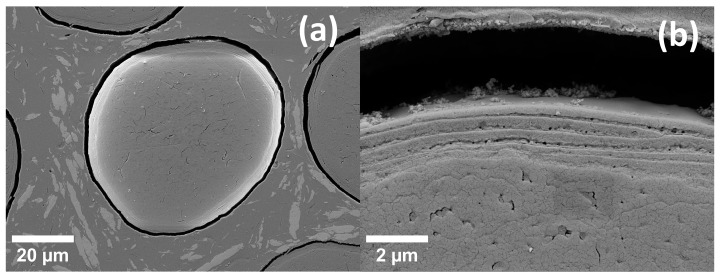
SEM micrographs of the cross-section with the cuticle visible as concentric rings, (**a**) an overview, and (**b**) in detail. This is probably the origin of the SAXS lipid ring, which arises from ordered polar lipids. The gap in (**b**) arises from the shrinkage of the embedding resin.

In Figure 7, SEM micrographs are presented for hair initially before loading (Figure 7a,b) and for hair being subjected to 10% macroscopic strain (Figure 7c,d). Obviously, scales are aligned with the hair shaft and exhibit a smooth surface in the unstrained case, whereas scales deform and protrude from the hair shaft in the strained case. Moreover, at high strain, gaps form between the overlapping parts of the scales. The complete first strain cycle is also shown in the Supporting Information, Figure S10, and the strong effect of the strain in the second strain cycle after a waiting period of 100 min is shown in Figure S11. Concerning strain recovery, the effect of different waiting times is depicted in Figure 8. After straining the hair to 10%, the strain was relieved, and a micrograph of this hair after unloading and after the waiting period of 100 min is shown in Figure 8a,b. In Figure 8c,d, the same micrographs are presented for the longer waiting period of 1000 min. Though the scales are much smoother and more regularly arranged than in the strained case, in the upper figures for the shorter waiting period, scales are still deformed and protrude from the hair shaft, whereas they seem to be nearly completely aligned with the hair shaft after the longer waiting period. Their appearance after the longer waiting time is then closer to the initial unstrained hair. In the transverse cut in Figure 9, the cuticle is clearly seen as concentric rings. This is probably the origin of the SAXS lipid ring, which arises from ordered polar lipids.

## 4. Discussion

As hair is a biological tissue with an elongated shape and a hierarchical structure with different structural elements, one can expect that there is no unique strain from the nano- to the macroscale if hair is subjected to tensile load. This is supported by Figure 3, in which the strain in different structural features was evaluated and compared to the macrostrain, and the numerical values are given in Table 1. The strain in load direction is obtained from the meridional arc and originates from molecular structures along the IFs. As the IFs are well oriented along the long axis of hair, the distance change derived from this peak can be seen as a probe for the nanostrain in the IFs, as has already been proposed in the literature [21]. In our experiment, the nanostrain in the IFs is about 70% of the macrostrain, which is a very high value in comparison to other biological tissues with a hierarchical structure, such as collagen or bone, where a value of about 40% was found [27,28,29]. Also, the time for strain recovery (and maybe physical self-healing) is 75 s in human bone [32] but in the range of some tens of thousands of seconds in human hair (from the time to recover the original IF distance in Figure 4). One can only speculate about the reason and the structural origin of these differences, but certainly, hair has only (a multiple of) two strands, whereas collagen is a triple helix with a complex regular alignment of its fibrils according to the Hodge-Petruska model [38]. A further reason could be that the hierarchical levels are much more important for collagen and bone as they are living tissues with the possibility of cellular repair at different hierarchical levels, whereas hair is a dead tissue with excellent but predefined mechanical properties. Important for mechanics are also transverse properties: As the average distance of IFs also gives a strong X-ray signal, it is possible to measure the transverse deformation and to derive a “nanoscopic Poisson ratio ν_nano_” by dividing the relative distance change in the IFs (the transverse strain) by the relative distance change along the IFs (the longitudinal strain). A value of about ν_nano_ = 0.44 is obtained, taking the values from Table 1. A theoretical value for an ideal volume retaining material would be 0.5, and many soft materials, such as elastomers [36] and fiber networks [37], are close to this value. A lower value for the Poisson ratio of roughly 0.3 is found for many metals, whereas much lower values occur in ceramics and ceramic fibers, where a Poisson ratio of only 0.2 or even 0.1 was experimentally found [39]. Within the macrofibrils, the IFs are composed of a double-twist architecture in which a central IF is surrounded by concentric rings of tangentially angled IFs [18]. The matrix in the cortex is far from being amorphous [19] but has a grainy nanoscale structure in the size of 2–4 nm and is composed of keratin-associated proteins [19]. Even with advanced TEM research, the precise coupling of IFs is unknown, and we can only state from our results that mechanical properties such as the Poisson ratio are rather typical for soft matter.

**Figure 10:**
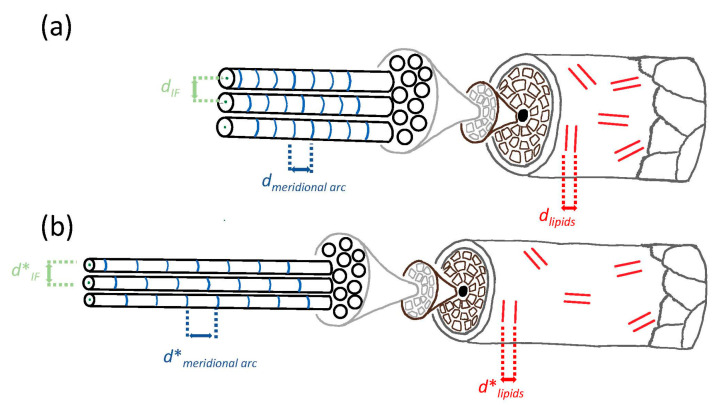
Scheme of deformation model of (**a**) unstrained and (**b**) strained hair. Oriented IFs (surrounded by a granular matrix) are highly oriented in the cortex and able to take up most of the strain, whereas lipids with a short-range order, visualized by two red lines, are preferably found in the cuticle. These lipids are not preferentially oriented and are exposed to a much lower strain due to the flexibility of the scales. The distances measured from the respective SAXS features are denoted by d_meridional arc_ for the meridional arc, d_IF_ for the IF distance, and d_lipids_ for the mean distance of lipid bilayers. The distances in the strained condition are indicated with an asterisk.

Obviously, from Table 1, the strain of the lipid bilayers is considerably smaller by even a factor of more than six for 10% macroscopic strain. This indicates that the bilayers do not take part in the deformation of hair to the same amount as the IFs. Nevertheless, the strain in the lipids in load direction and perpendicular to it (evaluated from the bilayer ring in Figure 1c, Table 1, as well as load cycles in Figure 6) gives a Poisson ratio at the nano level of the lipids of ν_lipids_ = 0.40, which is rather similar to the Poisson ratio in the cortex from the IFs of ν_nano_ = 0.44, taking the experimental uncertainties of the determination of a Poisson ratio into account. To visualize this deformation behavior, Figure 10 shows a model of the IFs and the surrounding matrix as the main structural element of the cortex. The IFs are highly oriented and able to take up most of the strain, whereas the lipids with a short-range order are mainly present in the cuticle, without preferred orientation, and exposed to a much lower strain due to the flexibility of the scales. It cannot be excluded that the lipid clusters do not deform due to a weak interaction and lose connection with the surrounding structure, and this possibility was already considered in [40]. However, since the observed lipid rings found in the SAXS patterns are caused by their lamellar arrangement [24,41], it is more probable that the region where the lipids are in the hair does not deform equally as the IFs in the cortex. This is supported by the SEM micrographs in Figure 7, where, under strain, the scales protrude from the hair surface and become partly detached (clearly visible in Figure 7d). Gaps form between the scales, which strongly indicates that the scales and the cuticle deform to a much smaller amount than the cortex of the hair. That the cuticle is richer in lipids than the cortex was directly proven by Fourier transform infrared synchrotron spectroscopy using the intensity of the lipid characteristic frequency at 2919 cm^−1^ [26], but differences for ethnic hair types (Caucasian, Asian, African) were observed. Using confocal microscopy on hair with transversal cuts dyed with Nile red, it was possible to distinguish between different types of lipids, and a very polar arrangement of lipids in the cuticle was found [40]. Due to the polarity, there is a higher probability for a short-range order of the lipid layers, which gives an intensity increase in the SAXS pattern at the typical distance due to the increase in the structure factor. We therefore conclude that the lipid ring arises from the lipids with a regular arrangement, i.e., exhibiting a short-range order, and these lipids are mainly located in the cuticle. Again, one cannot exclude that a specific amount of disordered (amorphous) lipids is also present in the cortex, but these lipids would give a constant scattering contribution and act, therefore, as background in the integrated SAXS patterns.

Strain recovery of the IF distances in SAXS was observed in Figure 4 between 100 and 1000 min, i.e., within a day. The effect is also visible along the long axis of the IFs in the load direction (Figure 5) but less pronounced than in the transverse direction. To investigate this time dependence, a load program similar to SAXS experiments was performed for hair in the SEM (applying 10% macrostrain and then varying the waiting period). This is seen in Figure 8, in which the hair sample after 100 min still shows some deformation and protrusion of the scales from the hair shaft, whereas, after an increase in the waiting time to 1000 min, the appearance of the hair sample is nearly identical to the initial unstrained hair in Figure 7a,b. Though we use SEM to look at the surface of hair at the microscale, and strain recovery was determined within the cortex at the nanoscale, it can be assumed that the return of the outer structure to its original shape is caused by the strain recovery of the inner structure. A possible cause for the observed recovery could be found in the structural model of Murthy et al. [23]. They proposed that the dimers form either a tetramer (protofilament), which builds up an open ring structure, or an octamer (protofiber), which forms a compact ring-core structure for the cross-section of the IF [23]. As the open structure allows easier twisting, this structure probably sustains a higher strain. If hair is subjected to large strains, tensile load could induce a switching between these two configurations, which can be reversed after some time. The effect would be a recovery of the structural and mechanical properties along the long axis of hair, coinciding with our observations.

## 5. Conclusions

From the evaluation of four features in the SAXS pattern during loading, we determined the deformation behavior of the IFs and measured a nanoscopic Poisson ratio of ν_nano_ = 0.44 and a Poisson ratio of the lipids of ν_lipids_ = 0.40. The deformation in the lipids is much lower than in the IFs, even by a factor of six at a macroscopic strain of 10%. Accompanying SEM experiments of unstrained and strained hair showed that the deformation of the scales in the cuticle of the hair is much lower than the deformation of the cortex, which leads to the conclusion that those lipids contributing to the X-ray intensities are in the cuticle. This coincides with results from synchrotron-assisted FTIR or confocal microscopy. Strain recovery was investigated with in operandi SAXS during two load cycles and with varying waiting times in between. Although strain recovery was rather fast for longitudinal extension of the IFs, transversal strain recovery in the matrix between the IFs required a time of some ten thousand seconds. After this period, the structure was identical to the initial structure. This suggests an interpretation that this is the typical time of self-healing for Caucasian human hair. This is supported by our SEM micrographs, which show that, after straining hair and waiting 1000 mins, strained hair appears to be nearly identical to the initial unstrained state. As a final remark, the deformation behavior of hair is complex, but surprisingly, more levels of deformation are found for the strain in the transverse direction across the cross-section than for the strain in the (loaded) longitudinal direction.

## Figures and Tables

**Table 1:** Numerical values for the four structural features evaluated in Figure 3: Mean values and standard error (only the statistical error from the six initial unstrained hair bundles) of fits for the IF distance, the meridional arc, and the lipid ring in and perpendicular to load direction. ${\bar{d}}_{0}$ is the mean initial distance in real space in nm, and the mean relative changes $\bar{∆d/d_{0}}$ of the distances at 2% and 10% macroscopic strain are given in percent.

	$\bar{d_{0}}$/nm	$\bar{{∆d/d}_{0}}$ in % for 2% Macrostrain	$\bar{{∆d/d}_{0}}$ in % for 10% Macrostrain
IF distance	8.924 ± 0.029	−0.99 ± 0.18	−3.10 ± 0.30
Meridional arc	6.583 ± 0.026	1.66 ± 0.58	7.00 ± 0.43
Lipid peak in load direction	4.597 ± 0.013	0.67 ± 0.09	1.12 ± 0.11
Lipid peak Perpendicular to load direction	4.598 ± 0.015	−0.18 ± 0.05	−0.43 ± 0.08

## Data Availability

The data that support the findings of this study are available from the corresponding author upon reasonable request.

## Supplementary Materials

The following Supporting Information can be downloaded https://www.mdpi.com/article/10.3390/life13122246/s1, Figure S1: Description of equipment. Figure S2: SAXS image with integration sectors. Figure S3: Background subtraction. Figure S4: Example for the fit of IF peak and meridional arc. Figure S5: Example for the fit of lipid ring in and perpendicular to load direction. Figure S6: Distance change between IFs for 2% macrostrain. Figure S7: Distance change for meridional arc for 2% macrostrain. Figure S8: Distance change in lipid bilayers for 2% macrostrain. Figure S9: Distance change in lipid bilayers after multiple strain cycles of 10% macrostrain. Figure S10: SEM micrographs unstrained, strained, and unstrained after a waiting period of 100 min. Figure S11: SEM micrographs of strongly deformed scales, second strain cycle [42].

## References

[1] Gottschaldt K.-M., Iggo A., Young D.W. (1973). Functional characteristics of mechanoreceptors in sinus hair follicles of the cat. J. Physiol..

[2] Metwally S., Comesana S.M., Zarzyka M., Szewczyk P.K., Karbowniczek J.E., Stachewicz U. (2019). Thermal insulation design bioinspired by microstructure study of penguin feather and polar bear hair. Acta Biomater..

[3] Catania K.C. (2008). No taming the shrew. Nat. Hist. Mag..

[4] Amador G.J., Hu D.L. (2015). Cleanliness is next to godliness: Mechanisms for staying clean. J. Exp. Biol..

[5] Popescu C., Höcker H. (2007). Hair—the most sophisticated biological composite material. Chem. Soc. Rev..

[6] Morel O.J.X., Christie R.M. (2011). Current trends in the chemistry of permanent hair dyeing. Chem. Rev..

[7] Nohynek G.J., Fautz R., Benech-Kieffer F., Toutain H. (2004). Toxicity and human health risk of hair dyes. Food Chem. Toxicol..

[8] Aburjai T., Natseh F.M. (2003). Plants used in cosmetics. Phytother. Res..

[9] Müllner A.R.M., Pahl R., Brandhuber D., Peterlik H. (2020). Porosity at different structural levels in human and yak belly hair and its effect on hair dyeing. Molecules.

[10] Astbury T.W., Street A. (1931). X-ray studies of the structure of hair, wool, and related fibres. I.-General. Philos. Trans. R. Soc. Lond. A.

[11] Astbury T.W., Woods H.J. (1933). X-ray studies of the structure of hair, wool, and related fibres. II.-The molecular structure and elastic properties of keratin. Philos. Trans. R. Soc. Lond. A.

[12] Lee J., Kwon H.J. (2013). Measurement of stress-strain behaviour of human hair fibres using optical techniques. Int. J. Cosmet. Sci..

[13] Hu Z., Li G., Xie H., Hua T., Chen P., Huang F. Measurement of Young’s modulus and Poisson’s ratio of human hair using optical techniques. Proceedings of the Fourth International Conference on Experimental Mechanics.

[14] Fratzl P., Weinkamer R. (2007). Nature’s hierarchical materials. Progr. Mater. Sci..

[15] Fraser R.D.B., Macrae T.P., Parry D.A.D., Suzuki E. (1986). Intermediate filaments in α-keratins. Proc. Natl. Acad. Sci. USA.

[16] Parry D.A.D., Steinert P.M. (1999). Intermediate filaments: Molecular architecture, assembly, dynamics and polymorphism. Q. Rev. Biophys..

[17] Tintner J., Rennhofer H., Kennedy C.J., Revie W.A., Weber H., Pavlik C., Lanszki J. (2020). Recalcitrance of hair in historical plasters. Polym. Degrad. Stab..

[18] Harland D.P., Walls R.J., Vernon J.A., Dyer J.M., Woods J.L., Bell F. (2014). Three-dimensional architecture of macrofibrils in the human scalp hair cortex. J. Struct. Biol..

[19] Kair M., Wang X., Zhu B., Liu J., Harland D., Popescu C. (2017). The structure of the “amorphous” matrix of keratins. J. Struct. Biol..

[20] Briki F., Busson B., Doucet J. (1998). Organization of microfibrils in keratin fibers studied by X-ray scattering: Modelling using the paracrystal concept. Biochim. Biophys. Acta.

[21] Kreplak L., Franbourg A., Briki F., Leroy F., Dallé D., Doucet J. (2002). A new deformation model of hard α-keratin fibers at the nanometer scale: Implications for hard α-keratin intermediate filament mechanical properties. Biophys. J..

[22] Rafik M.E., Doucet J., Briki F. (2004). The intermediate filament architecture as determined by X-ray diffraction modeling of hard α-keratin. Biophys. J..

[23] Murthy N.S., Wang W., Kamath Y. (2019). Structure of intermediate filament assembly in hair deduced from hydration studies small-angle neutron scattering. J. Struct. Biol..

[24] Coderch L., Méndez S., Barba C., Pons R., Martí M., Parra J.L. (2008). Lamellar rearrangement of internal lipids from human hair. Chem. Phys. Lipids.

[25] Kreplak L., Briki F., Duvault Y., Doucet J., Merigoux C., Leroy F., Lévêque J.L., Miller L., Carr G.L., Williams G.P. (2001). Profiling lipids across caucasian and afro-american hair transverse cuts, using synchrotron infrared microspectrometry. Int. J. Cosmet. Sci..

[26] Barba C., Oliver M.A., Martí M., Kreuzer M., Coderch L. (2022). Lipid distribution on ethnic hairs by Fourier transform infrared synchrotron spectroscopy. Ski. Res. Technol..

[27] Fratzl P., Misof K., Zizak I., Rapp G., Amenitsch H., Bernstorff S. (1997). Fibrillar structure and mechanical properties of collagen. J. Struct. Biol..

[28] Gupta H.S., Seto J., Wagermaier W., Zaslansky P., Boesecke P., Fratzl P. (2006). Cooperative deformation of mineral and collagen in bone at the nanoscale. Proc. Natl. Acad. Sci. USA.

[29] Gupta H.S., Wagermaier W., Zickler G.A., Raz-Ben Aroush D., Funari S.S., Roschger P., Wagner H.D., Fratzl P. (2005). Nanoscale deformation mechanisms in bone. Nano Lett..

[30] Fantner G.E., Hassenkam T., Kindt J.H., Weaver J.C., Birkedal H., Pechenik L., Cutroni J.A., Cidade G.A.C., Stucky G.D., Morse D.E. (2005). Sacrificial bonds and hidden length dissipates energy as mineralized fibrils separate during bone fracture. Nat. Mater..

[31] Gupta H.S., Fratzl P., Kerschnitzki M., Benecke G., Wagermaier W., Kirchner H.O.K. (2007). Evidence for an elementary process in bone plasticity with an activation enthalpy of 1 eV. J. R. Soc. Interface.

[32] Akbarzadeh J., Puchegger S., Stojanovic A., Kirchner H.O.K., Binder W.H., Bernstorff S., Zioupos P., Peterlik H. (2014). Timescales of self-healing in human bone tissue and polymeric ionic liquids. Bioinspired Biomim. Nanobiomater..

[33] Campanella A., Döhler D., Binder W.H. (2018). Self-healing in supramolecular polymers. Macromol. Rapid Commun..

[34] Zare P., Stojanovic A., Herbst F., Akbarzadeh J., Peterlik H., Binder W.H. (2012). Hierarchically nanostructured polyisobutylene-based ionic liquids. Macromolecules.

[35] Schneider M.R., Schmidt-Ullrich R., Paus R. (2009). The hair follicle as a dynamic miniorgan. Curr. Biol..

[36] Javanmardi Y., Colin-York H., Szita N., Fritzsche M., Moeendarbary E. (2021). Quantifying cell generated forces: Poisson’s ratio matters. Commun. Phys..

[37] Picu R.C. (2011). Mechanics of random fiber networks—A review. Soft Matter.

[38] Petruska J.A., Hodge A.J. (1964). A subunit model for the tropocollagen macromolecule. Proc. Natl. Acad. Sci. USA.

[39] Villeneuve J.F., Naslain R., Fourmeaux R., Sevely J. (1993). Longitudinal/radial thermal expansion and Poisson ratio of some ceramic fibres as measured by transmission electron microscopy. Compos. Sci. Technol..

[40] Cruz C.F., Fernandes M.M., Gomes A.C., Coderch L., Martí M., Méndez S., Gales L., Azoia N.G., Shimanovich U., Cavaco-Paulo A. (2013). Keratins and lipids in ethnic hair. Int. J. Cosmet. Sci..

[41] Yang F.C., Zhang Y., Rheinstädter M.C. (2014). The structure of people’s hair. PeerJ.

[42] Tchakalova V., Oliveira C.L.P., Neto A.M.F. (2023). New lyotropic complex fluid structured in sheets of ellipsoidal micelles solubilizing fragrance oils. ACS Omega.

