# Supplementary material for: Strain and Strain Recovery of Human Hair from the Nano- to the Macroscale
Source: Life (Basel). 2023 Nov 22;13(12):2246. doi: 10.3390/life13122246 (PMC10744986; doi:10.3390/life13122246)
Supplement: Supplementary file 1 [file life-13-02246-s001.zip › life-2665476-supplementary.pdf]

Supporting Information: Strain and Strain Recovery of Human Hair from the Nano- to the Macroscale

Brigitte Waldmann[1], Martin F. T. Hassler [1,2], Alexander R. M. Müllner [1,2], Stephan Puchegger [1] and Herwig Peterlik [1,*]

1 Faculty of Physics, University of Vienna, Boltzmanngasse 5, 1090 Vienna, Austria; brigitte.waldmann@univie.ac.at (B.W.); martin.hassler@univie.ac.at (M.F.T.H.); alexander.muellner@univie.ac.at (A.R.M.M.); stephan.puchegger@univie.ac.at (S.P.)
2 Vienna Doctoral School in Physics, University of Vienna, Boltzmanngasse 5, 1090 Vienna, Austria
* Correspondence: herwig.peterlik@univie.ac.at

1. Description of equipment and evaluation procedure

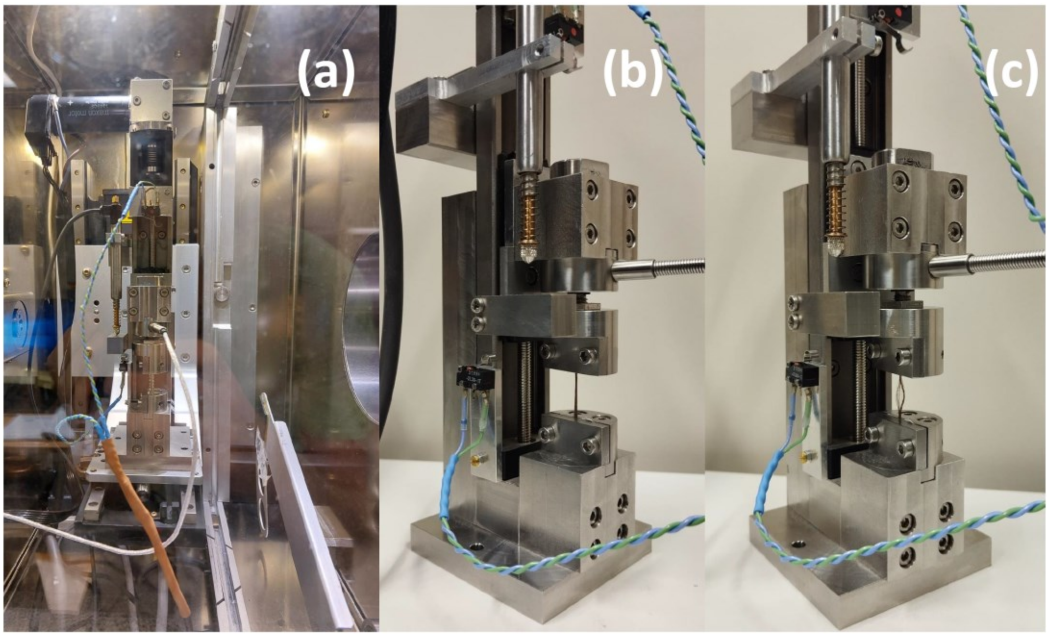

Figure S1. Tension test apparatus with stepper motor and two ways to determine the position (step counter and linear variable differential transducer). (**a**) Built into SAXS equipment (Nanostar, Bruker AXS), (**b**) with strained hair bundle, (**c**) with relaxed hair bundle as in the waiting period.

Figure S2 shows a typical 2D image with the integration sectors of ± 15 degrees around horizontal and vertical axes. The integration sectors are fixed and not variable as in [1], because the hair bundles are carefully aligned in the tension test equipment, which is in a fixed position in the SAXS equipment. Azimuthal integration was performed in each sector to obtain the scattering intensities in dependence on scattering vector q. Each of the sectors was then evaluated and the horizontal (perpendicular to load direction) and the vertical (in load direction) fit parameters were averaged.

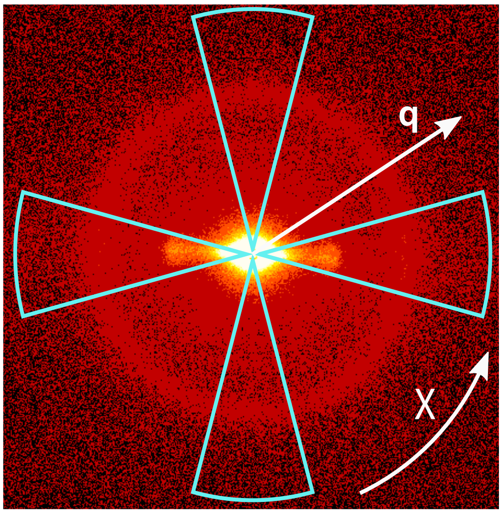

Figure S2. Showing a typical 2D image with the integration sectors.

The background was subtracted with a power law function $I_{bgr}=I_0\ q^{-d}$ for each sector. The mean value of the exponent was d=2.56±0.10, in accordance with the literature (d=2.55 in [2], d=2.33 in [3]).

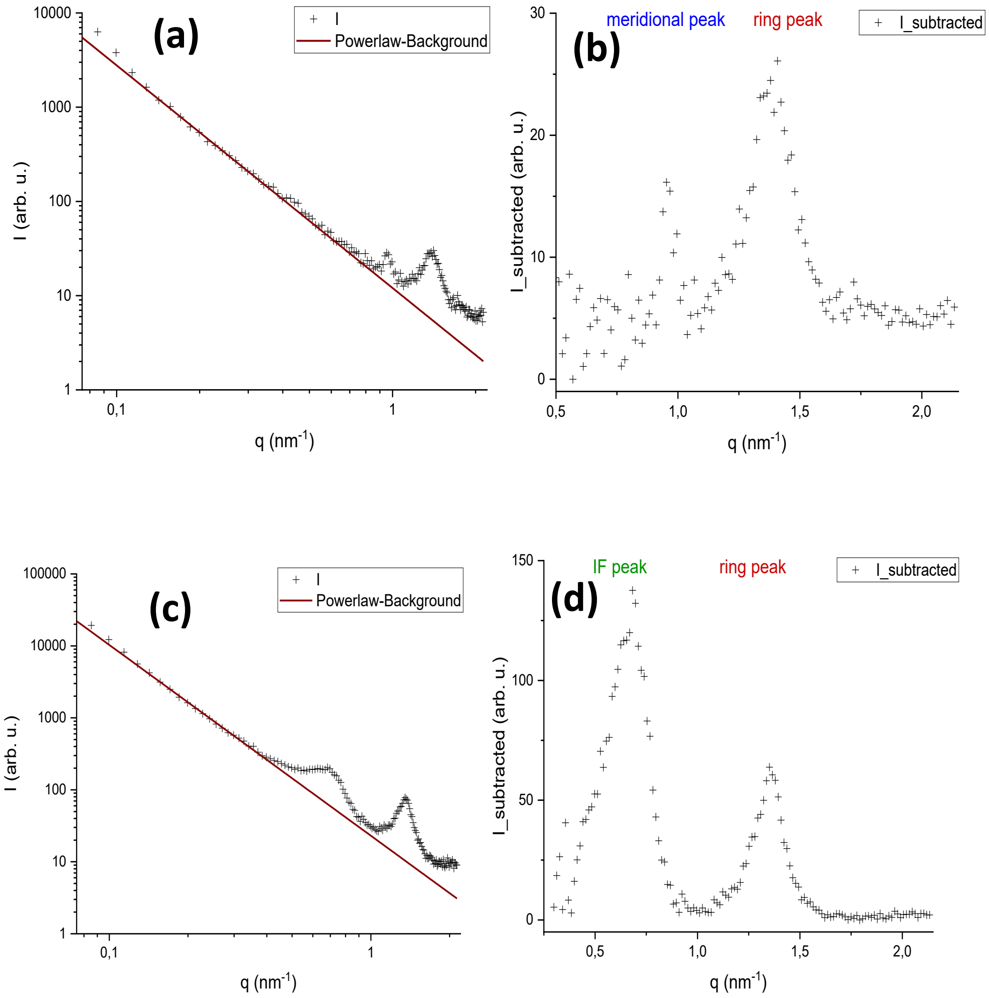

Figure S3. (**a**) Integrated data from vertical sector and power law subtraction. (**b**) Vertical sector, subtracted intensity with meridional and lipid ring peak. (**c**) Integrated data from horizontal sector and power law subtraction. (**d**) Horizontal sector, subtracted intensity with IF peak and ring peak.

2. Description of fit procedure

To take the specific shape of each peak into account and to increase the precision, a separate fit function or procedure was used for each peak. The IF peak was fitted with eq (S1), a skew normal distribution f(q) with a constant y_0, an amplitude A, a location parameter q_c, a scale parameter ω and a shape parameter α. The fit interval were all datapoints around the intensity maximum I_0 with an intensity $I > I_0/4$. The maximum of the curve was then determined numerically from the fit. Figures S4a and S4b show an unstrained and strained hair bundle with the fit curves.

$$f(q) = y_0 + \frac{A}{\sqrt{2\pi}\,\omega}\exp\left(-\frac{(q-q_c)^2}{2\omega^2}\right)\cdot\left[1+\operatorname{erf}\left(\alpha\cdot\frac{q-q_c}{\omega\sqrt{2}}\right)\right]. \tag{S1}$$

The lipid ring peaks were fitted with a Lorentzian function, eq. (S2), with a constant y_0, an amplitude A, a location parameter q_c, and a width parameter w, the half-width at half-maximum. The fit interval were all datapoints around the intensity maximum I_0 with an

intensity $I > I_0/3$. The maximum of the curve is the location parameter q_c. Figures S5a and S5b show an unstrained and strained hair bundle with the fit curves for the lipid ring in load direction, Figures S5c and S5d show the respective figures for the unstrained and strained hair bundle perpendicular to load direction

$$f(q) = y_0 + \frac{2A}{\pi}\frac{w}{4\,(q - q_c)^2 + w^2} \quad . \tag{S2}$$

The meridional arc peaks were calculated from eq. (S3), the center of gravity, for a symmetric interval around the intensity maximum (± 10 points, in total 21 points). Figures S4c and S4d show the figures for an unstrained and strained hair bundle with the points in blue were taken for the calculation.

$$q_m = \frac{\sum_k q_k\, I(q_k)}{\sum_k I(q_k)} \ . \tag{S3}$$

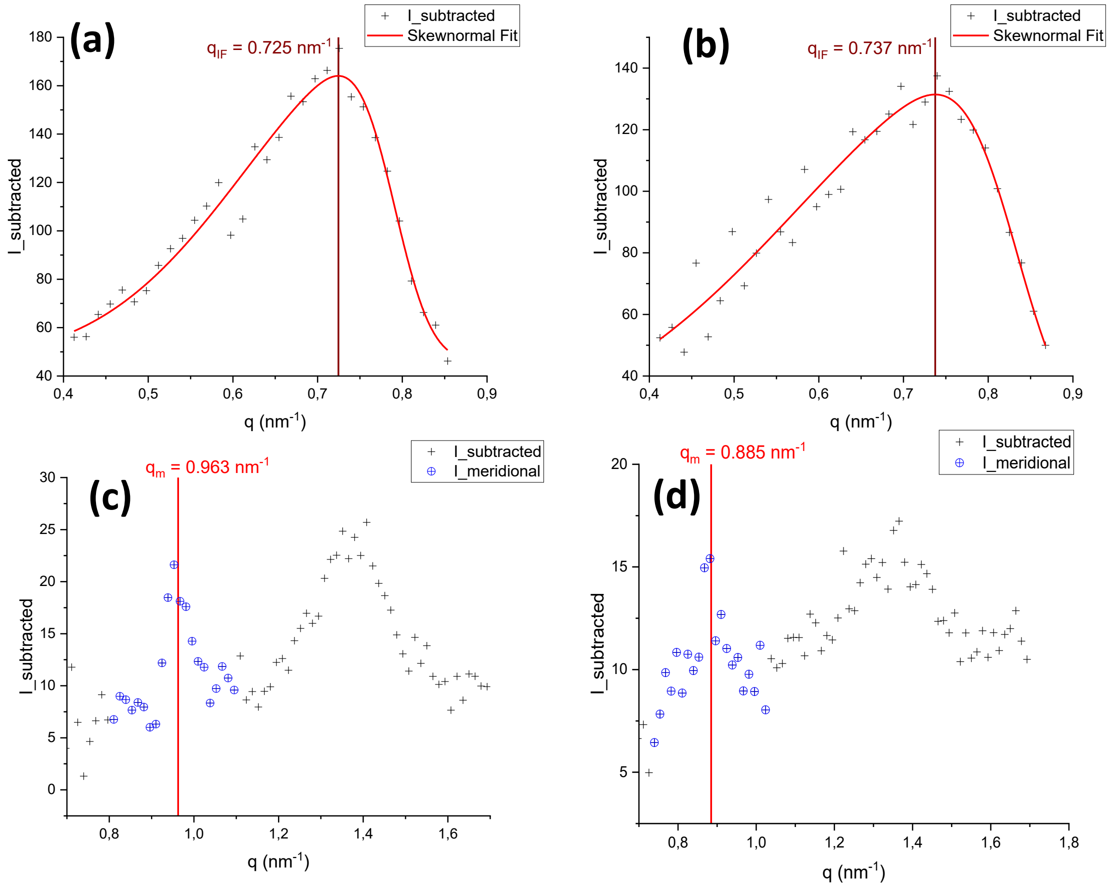

Figure S4. Fit with a skew normal function of IF peak of a hair bundle, (**a**) unstrained, (**b**) strained, with a shift towards larger q-values, i.e. smaller distances in real space. Center of gravity of a meridional arc peak, (**c**) unstrained, (**d**) strained, with a shift towards smaller q-values, i.e. larger distances in real space.

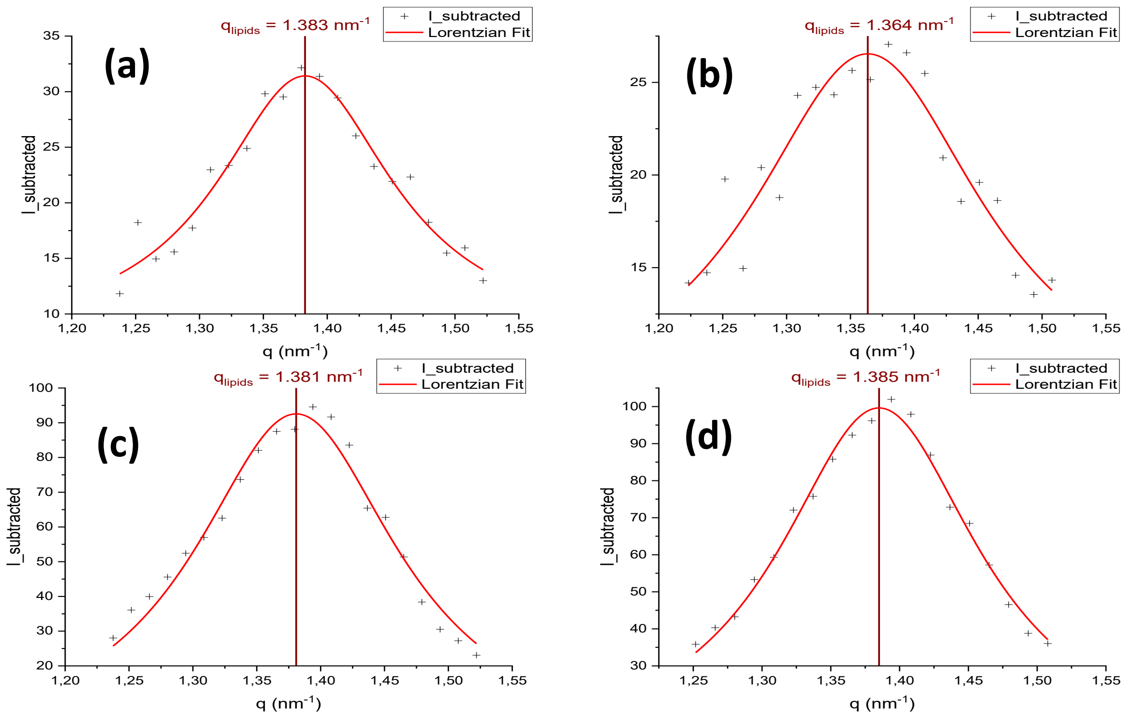

Figure S5. Fit with a Lorentzian function of lipid ring peaks of a hair bundle in load direction, (**a**) unstrained, (**b**) strained, with a shift towards smaller q-values, i.e. larger distances in real space, and perpendicular to load direction, (**c**) unstrained, (**d**) strained, with a shift towards larger q-values, i.e. smaller distances in real space.

3. Deformation at 2% macrostrain

Figures S6 to S8 show the data for 2% macroscopic strain, Figure S6 for the distance of the intermediate filaments (IFs), Figure S7 for the distance from axially staggered molecules along the length direction of the IFs, evaluated from the meridional arc, and Figure S8 for the lipid bilayer distance in and perpendicular to load direction. The sample was subjected to multiple load/strain cycles with 2% strain and varying the waiting period between the load cycles (10 min, 10^2 min, 10^3 min, 10^4 min) to study strain recovery at the nanoscale

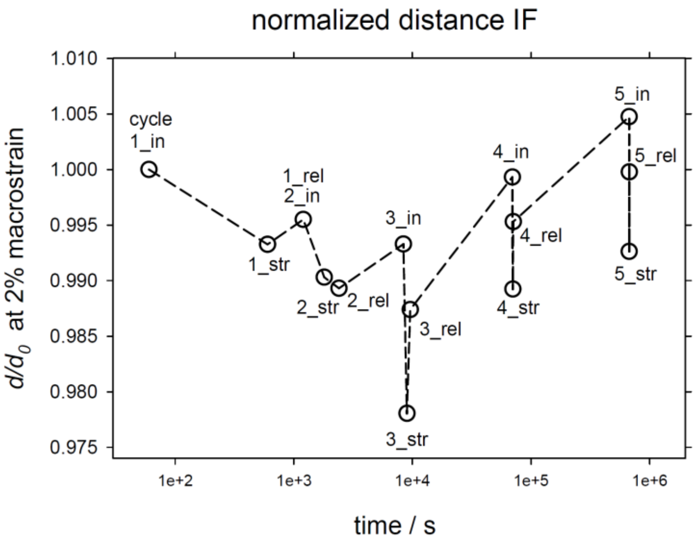

Figure S6. Change of distances between IFs, normalized to initial value, at 2%macrostrain. One sample was strained multiple times, with the number indicating the load/strain cycle and the

index the moments, when SAXS patterns were recorded to measure the IF distance: _in, initial distance, _str, distance at maximum strain, _rel, distance after strain release to zero. The waiting period was 10 min for cycle 2, 10^2 min for cycle 3, 10^3 min for cycle 4 and 10^4 min for cycle 5. The dashed lines are for better visualization of the strain cycles.

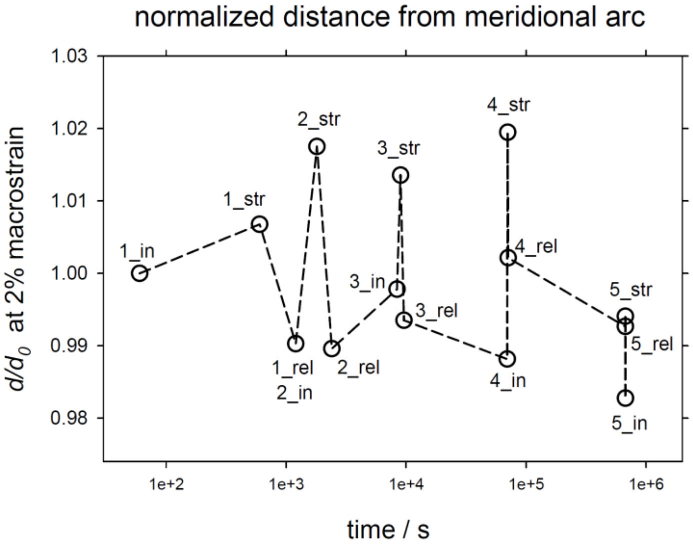

Figure S7. Change of distances from molecule staggering along the IFs, normalized to initial value, at 2% macrostrain. One sample was strained multiple times, with the number indicating the load/strain cycle and the index the moments, when SAXS patterns were recorded to measure the molecule staggering distance: _in, initial distance, _str, distance at maximum strain, _rel, distance after strain release to zero. The waiting period was 10 min for cycle 2, 10^2 min for cycle 3, 10^3 min for cycle 4 and 10^4 min for cycle 5. The dashed lines are for better visualization of the strain cycles.

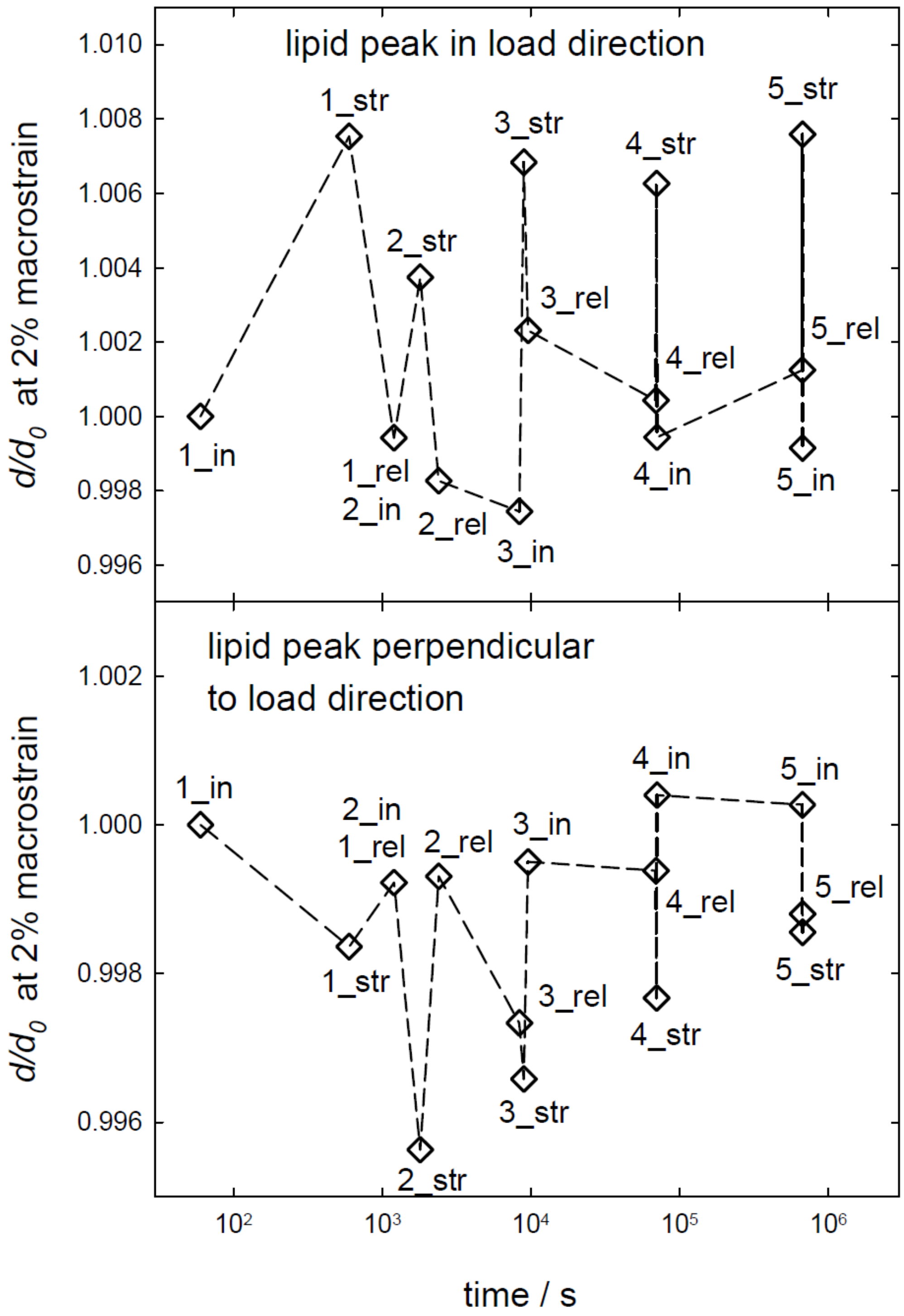

Figure S8. Change of lipid bilayer distance in load direction (upper figure) and perpendicular to load direction (lower figure) for 2% macrostrain. Both distances are normalized to initial value. One sample was strained multiple times, with the number indicating the load/strain cycle and the index the moments, when SAXS patterns were recorded to measure the lipid bilayer distance: _in, initial distance, _str, distance at maximum strain, _rel, distance after strain release to zero. The waiting period was 10 min for cycle 2, 10^2 min for cycle 3, 10^3 min for cycle 4 and 10^4 min for cycle 5. The dashed lines are for better visualization of the strain cycles.

4. Deformation of lipid peak for multiple strain cycles at 10% macrostrain

Figure S9 is similar to Figure S8 and shows the lipid bilayer distance for the strain cycles with different waiting times in and perpendicular to load direction, but for a macroscopic strain of 10%.

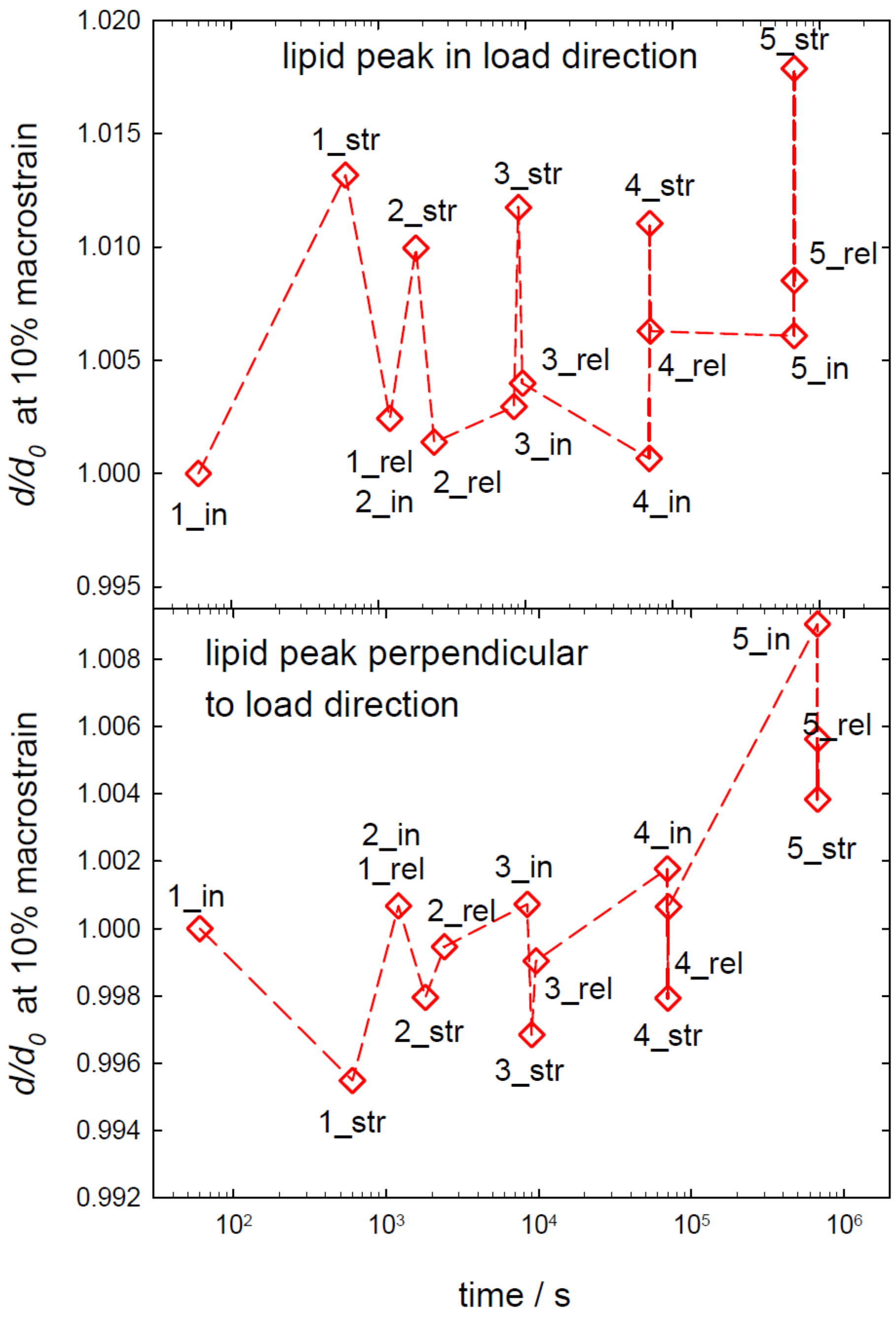

Figure S9. Change of lipid bilayer distance in load direction (upper figure) and perpendicular to load direction (lower figure) for 10% macrostrain. Both distances are normalized to initial value. One sample was strained multiple times, with the number indicating the load/strain cycle and the index the moments, when SAXS patterns were recorded to measure the lipid bilayer distance: _in, initial distance, _str, distance at maximum strain, _rel, distance after strain release to zero. The waiting period was 10 min for cycle 2, 10^2 min for cycle 3, 10^3 min for cycle 4 and 10^4 min for cycle 5. The dashed lines are for better visualization of the strain cycles.

5. SEM micrographs for unstrained initial state, at 10% macrostrain and unstrained after 100 min

Figure S10 shows SEM micrographs of the first strain cycle: (a) unstrained initial state, (b) with 10% strain, (c) unstrained after strain relief and a waiting period of 100 min. The original surface structure of (a) is nearly recovered in (c), with some small protrusions of scales from the surface.

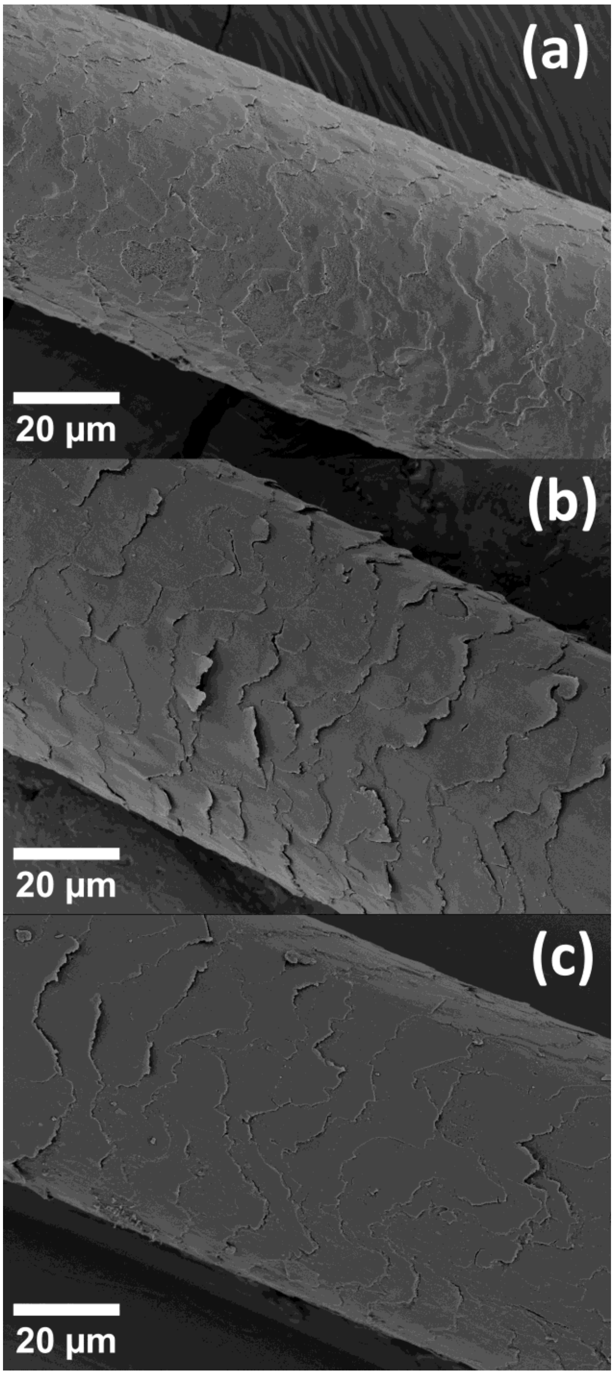

Figure S10. SEM micrographs of the first strain cycle: (**a**) unstrained initial state, (**b**) with 10% strain, (**c**) unstrained after strain relief and a waiting period of 100 min.

Figure S11 shows SEM micrographs of the second strain cycle in the 10% strained state after a waiting period of 100 min between the strain cycles, visualizing the strongly deformed and partly detached scales, (a) an overview, (b) in detail.

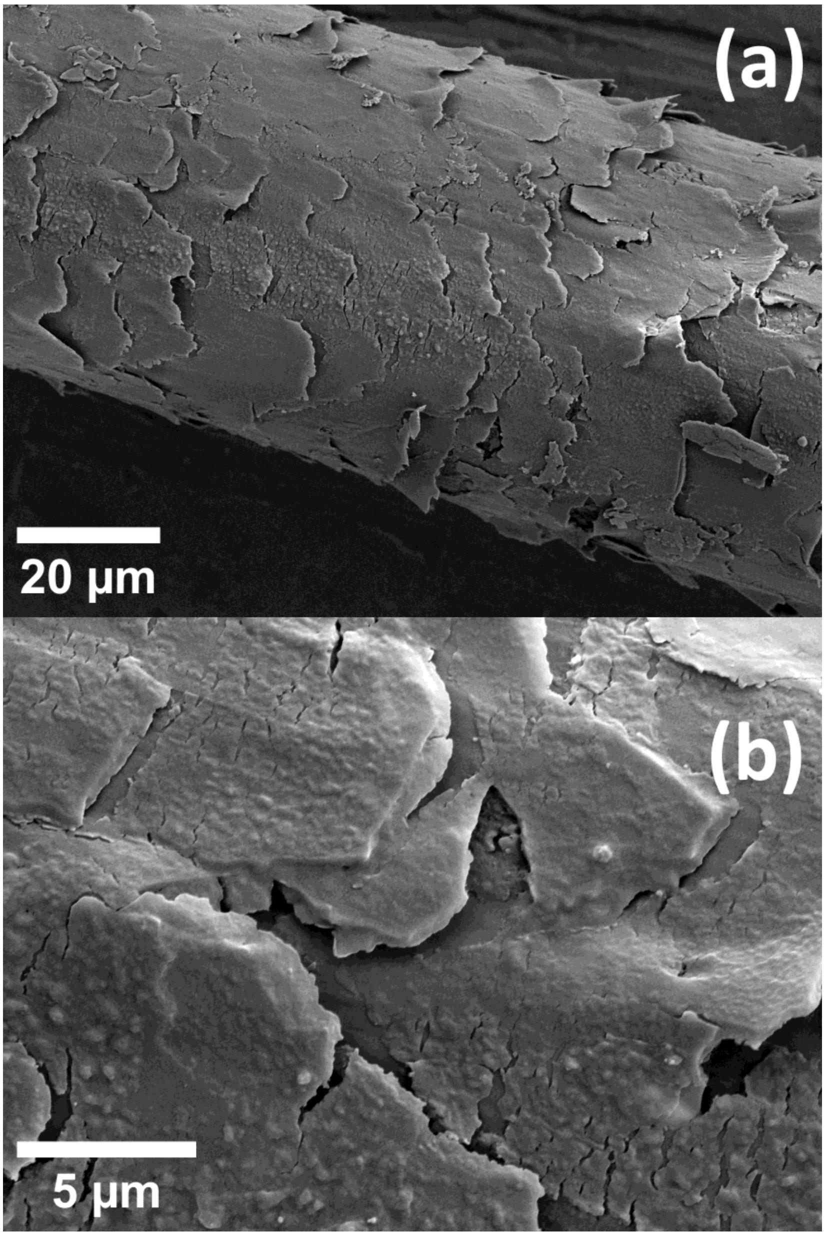

Figure S11. SEM micrographs of the second strain cycle after a waiting period of 100 min between the strain cycles in the 10% strained state. (**a**) overview, (**b**) detail, both visualizing strongly deformed scales, which are protruding from the surface and have shifted, as the cortex deforms significantly more than the cuticle and the scales.

References

1. Tchakalova, V., Oliveira, C.L.P., Neto, A.M.F. New lyotropic complex fluid structured in sheets of ellipsoidal micelles solubilizing fragrance oils. ACS Omega **2023**, 32, 29568-29584.
2. Murthy, N.S.; Wang, W.; Kamath, Y. Structure of intermediate filament assembly in hair deduced from hydration studies small-angle neutron scattering: Journal of Structural Biology **2019**, 206, 295-304.
3. Briki, F.; Busson, B.; Doucet, J. Organization of microfibrils in keratin fibers studied by X-ray scattering: Modelling using the paracrystal concept. Biochimica Biophysica Acta **1998**, 1429, 57–68.
